# Mental workload and driving

**DOI:** 10.3389/fpsyg.2014.01344

**Published:** 2014-12-02

**Authors:** Julie Paxion, Edith Galy, Catherine Berthelon

**Affiliations:** ^1^Laboratory of Accident Mechanism Analysis, French Institute of Science and Technology for TransportSalon-de-Provence, France; ^2^Research Center in the Psychology of Cognition, Language and Emotion, Aix-Marseille UniversityAix-en-Provence, France

**Keywords:** subjective workload, objective workload, driving performance, situation complexity, experience

## Abstract

The aim of this review is to identify the most representative measures of subjective and objective mental workload in driving, and to understand how the subjective and objective levels of mental workload influence the performance as a function of situation complexity and driving experience, i.e., to verify whether the increase of situation complexity and the lack of experience increase the subjective and physiological levels of mental workload and lead to driving performance impairments. This review will be useful to both researchers designing an experimental study of mental workload and to designers of drivers’ training content. In the first part, we will broach the theoretical approach with two factors of mental workload and performance, i.e., situation complexity and driving experience. Indeed, a low complex situation (e.g., highways), or conversely a high complex situation (e.g., town) can provoke an overload. Additionally, performing the driving tasks implies producing a high effort for novice drivers who have not totally automated the driving activity. In the second part, we will focus on subjective measures of mental workload. A comparison of questionnaires usually used in driving will allow identifying the most appropriate ones as a function of different criteria. Moreover, we will review the empirical studies to verify if the subjective level of mental workload is high in simple and very complex situations, especially for novice drivers compared to the experienced ones. In the third part, we will focus on physiological measures. A comparison of physiological indicators will be realized in order to identify the most correlated to mental workload. An empirical review will also take the effect of situation complexity and experience on these physiological indicators into consideration. Finally, a more nuanced comparison between subjective and physiological measures will be established from the impact on situation complexity and experience.

## INTRODUCTION

A driving situation is defined as the human-machine system environment (driver–vehicle) from the driver’s point of view. That represents a delimited section that ends with an environmental change (e.g., “free driving” turns over into “following”; Fastenmeier and Gstalter, 2007). The complexity of a driving situation depends on several elements that make up the environment, i.e., road design (motorways vs. rural roads vs. city roads), road layout (straight vs. with curves, even vs. inclined, junction vs. no junction) and traffic flow (high density vs. low density). This taxonomy of the situation complexity (Fastenmeier, 1995; Fastenmeier and Gstalter, 2007) has thus categorized a very complex situation as an urban road, with curves or junctions, and with a high traffic density. These elements characterizing a situation have to be taken into account to perform the driving task. For instance, drivers have to stop their vehicle in front of a stop sign at a junction. Therefore, sequential units of “driving tasks” correspond to “driving situations” (road sections).

Michon (1985) and de Waard (1996) has identified the driving activity with a hierarchy of tasks on three levels. The first level is strategic and constitutes the decision-making (e.g., choosing to follow a route). The second level is tactical and includes reactions or maneuvers faced to the situation (e.g., reactions to the other drivers’ behavior and maneuvers to follow the road). The third level is operational and concerns the vehicle control (e.g., managing the trajectory). These authors have identified a controlled or automatic processing of the information depending on the task level. For the first and the second levels, high-level processes are made with a slow, serial, conscious, and flexible controlled processing. Indeed, the decision-making and the maneuvers imply a voluntary processing of the different elements of the driving situation. The third level rather requires low-level processes, with a fast, unconscious and rigid automatic processing (Schneider and Shiffrin, 1977). For instance, sequences of actions to maintain the vehicle on the path are mainly automatic. However, this routine automation is only acquired with driving experience, which could explain the over-representation of young novice drivers in road accidents (Williams, 2003). Therefore, the situation complexity and the driving experience should influence the mode of processing that implies different levels of mental workload, with a controlled processing rather more costly than an automatic one. This question is particularly important in the driving context inasmuch as human errors, and more precisely mental workload related problems, are responsible for the majority of road accidents (Dijksterhuis et al., 2011).

Mental workload can be subjectively felt by the individual who perceives a cost while realizing a task (Hart and Staveland, 1988). In driving, we will explore which questionnaires are the most appropriate to reflect the subjective level of mental workload. Moreover, we will also examine how the situation complexity and the driving experience influence this subjective level of mental workload.

Mental workload can also be correlated to physiological modifications due to “the interaction of the task demands, the circumstances under which it is performed, and the skills, behaviors, and perceptions of the individual” (DiDomenico and Nussbaum, 2008, p. 977). Physiological indicators are thus often used as an objective measure of mental workload. The performance of a secondary task while driving can also be taken into consideration for an objective assessment of mental workload. However, as the core of this article is based on questionnaires and physiological indicators, the performance measure related to the dual-task will mainly be described to confirm whether the driving task is automated or not. We will thus examine which physiological measures are the most representative of mental workload, and how situation complexity and driving experience influence these physiological measures.

**FIGURE 1:**
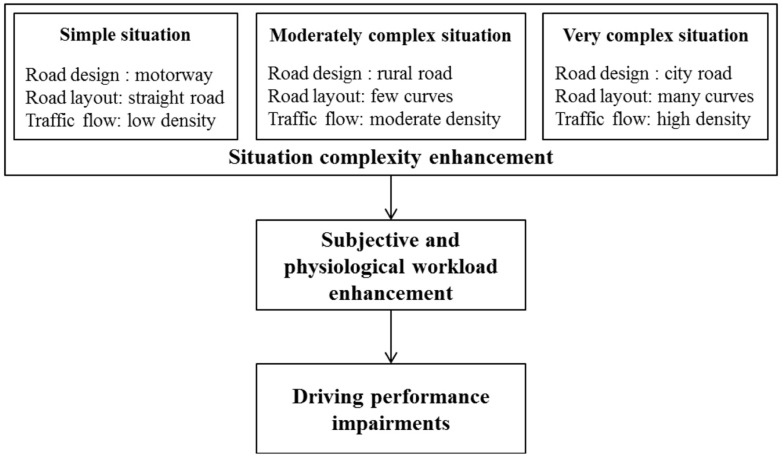
**Predictors of mental workload and performance impairments**.

Finally, we will seek to understand whether road accidents are due to a high level of mental workload measured subjectively, or measured physiologically, or both measured subjectively and physiologically. Additionally, we will here attempt to verify whether the effect of situation complexity and driving experience on performance is mediated by subjective and physiological mental workload (see **Figure 1**). Thus, we will try to answer to the following questions: do an increase of situation complexity and a lack of experience enhance the subjective and physiological levels of mental workload? In which case an increase of mental workload level leads to performance impairments? Does subjective and objective mental workload vary in the same way for novices and experienced drivers? Do they overestimate or underestimate their physiological state depending on situation complexity and on their driving experience? Finally, are the drivers’ behaviors more influenced by their subjective feeling, by their physiological mental state, or by both?

First, we will broach the theoretical approach about the influence of situation complexity and experience on mental workload and driving performance. Second, we will draw attention to the subjective measures that are mainly used to assess the level of mental workload. Third, we will focus on the physiological measures that are correlated to mental workload. Finally, we will compare these subjective and physiological measures as a function of the situation complexity and the driving experience.

## MENTAL WORKLOAD AND PERFORMANCE: A THEORETICAL APPROACH

### EFFECT OF SITUATION COMPLEXITY

A driving situation constitutes the major determinant of the mental workload of drivers (Verwey, 2000). The model of Meister (1976) and de Waard (1996), often used in the domain of driving, establishes the relation between task demands and performance depending on mental workload. This model assumes that when the situation is low demanding (e.g., in long and monotonous highways), or conversely when the situation is high demanding (e.g., in town with much information to process), drivers are overloaded with an increase of workload leading to performance impairments. Indeed, in monotonous situations, the driving task corresponds to the operational level of the trajectory maintenance, with an automatic processing of the information that can lead to a vigilance decrement. Performing the task thus requires a high effort to keep awake. The very complex situations also provoke a high level of workload, as they mainly require strategies and maneuvers (tasks of the first and second levels, see Michon, 1985) that imply a controlled processing of the large amount of information. However, some authors consider that all the information cannot be simultaneously taken into account, the individual’s working memory being characterized by a limited capacity (Broadbent, 1958; Kahneman, 1973; Posner, 1978; Wickens, 1984; de Waard, 1996). The single resource model of mental workload (Moray, 1967; Ryu and Myung, 2005) thus indicates that each individual has a limited capacity of processing, as the mental activities share the same resources. According to this model, when the task demands increase, the central nervous system increases the supply of resources necessary to perform the task.

The multiple resources theory (Wickens, 1984; de Waard, 1996) explains the existence of different categories of resources determined as a function of the input modality, i.e., the information restitution (visual canal, auditory canal, etc.), the codes processing and the response execution. If several tasks require resources using the same canal, the mental workload increases. The capacity to simultaneously perform several tasks thus depends on the quantity and on the mode of processing imposed by each task. If the processing is automatic, the task requires few resources and it will be possible to simultaneously perform several tasks. Inversely, if the processing is controlled, the task requires many resources and it will be difficult to realize several tasks simultaneously. Therefore, the resources required in very complex situations can exceed the available resources, leading to an increase of workload and to performance impairments (Robert and Hockey, 1997), characterized by an inappropriate speed and precision in the task realization (Bruni, 1995; Hadj-Mabrouk et al., 2001). Human errors can thus occur (Smiley and Brookhuis, 1987; de Waard, 1996). However, when the situation is moderately demanding, as in rural roads, the level of workload is relatively low and even if it goes up, compensatory strategies are set up in order to maintain a good performance (Meister, 1976; de Waard, 1996). Indeed, the driving task is rather operational (third level) with an automatic processing of the information, and can also be tactical (second level) with some maneuvers implying a controlled processing. Therefore, as the task is probably not entirely automated, drivers do not need to produce an effort to keep alert. Moreover, as the task is not too complex, drivers probably do not need to provide a high effort to perform it.

In monotonous and very complex situations, mental workload should thus be too high to correctly perform the driving task. These findings can be nuanced by the level of experience.

### EFFECT OF EXPERIENCE

The necessary mental workload to perform the driving tasks is slightly linked to the learning process and to the experience acquisition (Engströme et al., 2003). Indeed, the level of experience can modulate the influence of the driving tasks on the mode of information processing (controlled vs. automatic). Novice drivers have a low level of task automation (Patten et al., 2006) as the automatic processing is progressively acquired with practice. Indeed, the skill rule knowledge (SRK) model (Rasmussen, 1984) indicates a succession of steps to acquire a controlled behavior. Therefore, the driving activity induces a high level of mental workload for novice drivers (Sweller, 1993; Sweller et al., 1998; Wickens and Hollands, 2000; Patten et al., 2006), then the cognitive and motor skills acquired with practice requires a lower level of mental workload (Rasmussen, 1980, 1987; Patten et al., 2006), and the level of mental workload becomes very low for experienced drivers (Schneider and Shiffrin, 1977). In simple and monotonous situations, the automatic processing induced by the driving task should be more observed for experienced drivers than for the novice ones. Conversely, in complex situations, the controlled processing induced by the strategies and maneuvers should be more observed for novice drivers than for the experienced ones. The same driving situation can thus induce a lower mental workload for experienced drivers than for novice drivers. Epidemiological studies thus show that young novice drivers have a risk of accident 2–4 times higher than experienced drivers (Triggs, 2004; Di Stasi et al., 2009). An explanation could be provided by the subjective safety model (Brown, 1989; De Craen et al., 2008) that reveals that the strategies of adaptation are set up as a function of the situation characteristics and of the drivers. They particularly depend on the degree of precision in the perception of the situation complexity, of the task demands and of the cognitive capacities (Kuiken and Twisk, 2001; Mitsopoulos et al., 2006; De Craen et al., 2008).

Moreover, novice drivers often have a wrong assessment of the situation and lately set up compensatory strategies (Brown et al., 1987; Brown and Groeger, 1988; Gregersen, 1995; Mayhew and Simpson, 1995; De Craen et al., 2008). Indeed, they are likely to drive faster than experienced drivers, even in complex situations in which they need more skills (Quimby and Watts, 1981; Engströme et al., 2003; De Craen et al., 2008). According to the optimism bias, they also have a tendency to overvalue their abilities and to undervalue their risk of accident (McKenna, 1993). Moreover, their visual strategies are less efficient and less flexible than those of the experienced drivers (Falkmer and Gregersen, 2001; Engströme et al., 2003). Indeed, the novices monitor more the first plan in front of the vehicle and whatever the situation complexity, this strategy remains rigid. Inversely, the task automation acquired with learning can be inappropriate in some driving situations, a flexible behavior being required in unusual and hazard situations. However, experienced drivers are able to adapt their strategy by increasing their horizontal research (Crundall and Underwood, 1998; Engströme et al., 2003; Patten et al., 2006). They can thus process more information, which is useful to maintain a good performance despite a high level of mental workload. They also have a cognitive readiness by anticipating and scheduling the situations already known, which is necessary to make an efficient decision, especially in complex situations (Cegarra and van Wezel, 2012).

Different subjective and physiological measures of mental workload are identified in driving experiments. In the following paragraphs, we first present the characteristics of the subjective measures and then the studies’ results about the effect of situation complexity and driving experience on the subjective level of mental workload and on driving performance.

## SUBJECTIVE MEASURES OF MENTAL WORKLOAD

### QUESTIONNAIRES

Seven criteria are identified to assess the subjective level of workload (Eggemeier et al., 1991; Rubio et al., 2004):

(1) Sensitivity: detecting the changes of task difficulty and task demands,
(2) Diagnosticity: identifying the changes in workload variations and the cause of these changes,
(3) Selectivity/validity: being sensitive only to differences in cognitive demands,
(4) Intrusiveness: not interfering with the primary task performance,
(5) Reliability: reflecting consistently the mental workload,
(6) Implementation requirements: including aspects such as time, instruments, and software for the collection and analysis of data, and
(7) Subject acceptability: referring to the subject’s perception of the validity and to the usefulness of the procedure.

These criteria are not always considered by the questionnaires that assess the subjective level of mental workload in driving. Moreover, although the questionnaire technique is rapidly set up (Rubio et al., 2004; Paubel, 2011), it does not reflect the mental workload variation during the task realization (Cegarra and Chevalier, 2008). Indeed, this technique is a post-rationalization and when the experimental session is long, a recall bias can appear with a forgetting about the participant’s state during the session (Manning et al., 2001; Paubel, 2011).

In driving, three questionnaires constituted by a multidimensional scale are often compared. The subjective workload assessment technique (SWAT; Reid and Nygren, 1988) comprises scales assessing different workload components, i.e., time load, mental effort load, and psychological stress load. Three levels of subjective workload are proposed for each scale: low, medium, and high. The workload profile (WP; Tsang and Velazquez, 1996) questionnaire is based on the Wickens’ (1987) multiple resources model. Participants have to estimate the proportion of attentional resources used immediately after having experienced a particular task across eight workload dimensions: perceptual/central processing, response selection and execution, spatial processing, verbal processing, visual processing, auditory processing, manual output, and speech output (Rubio et al., 2004). The definition of each dimension is given to the participants. For each task, they have to provide a number between 0 (no demand) and 1 (maximum demand) that represents the proportion of attentional resources used in each of the eight workload dimensions. Finally, the NASA task load index (TLX; Hart and Staveland, 1988) questionnaire comprises six combinations of relevant factors characterizing the subjective workload: mental demands (amount of mental and perceptual activity required), physical demands (amount of physical activity required), temporal demands (amount of pressure felt due to the rate at which the task elements occurred), own performance (successful assessment in doing the task required and satisfaction assessment in accomplishing it), effort (difficulty assessment in having to mentally and physically work to accomplish the level of performance) and frustration (assessment of different feelings: insecure, discouraged, irritated, stressed and annoyed vs. secure, gratified, content, relaxed and complacent during the task). A 20 points scale ranged from 0 = ‘very low’ to 20 = ‘very high’ is proposed for each dimension, except from the scale of the Own Performance dimension which is ranged from 0 = ‘success’ to 20 = ‘failure.’

Two more specific questionnaires are also used to respectively, assess mental effort and mental workload in driving activity. The Rating Scale Mental Effort (RSME; Zijlstra, 1993) is a one-dimensional scale that only measures the mental effort with a continuous vertical line. Nine labels disposed on a 15-cm line (a 0–150 point scale) are ranged from “absolutely no effort,” through “rather much effort” to “extreme effort”. Participants mark the line at one of the nine points. The driving activity load index (DALI; Pauzié, 1994) assesses the subjective mental workload due to a driving task. It is inspired by the NASA-TLX and comprises six subscales, each going from low to high demanding: (1) Effort of attention (attention required by the activity), (2) Visual demand (necessary for the activity), (3) Auditory demand (necessary for the activity), (4) Temporal demand (specific constraint due to timing demand when running the activity), (5) Interference (possible disturbance when simultaneously running the activity with any other supplementary task), and (6) Situation stress (level of constraints/stress while conducting the activity).

These questionnaires are often described or compared by authors as a function of the criteria that they take into account (see **Table 1**; Zijlstra, 1993; Pauzié, 1994; Olar and Deconde, 2004; Rubio et al., 2004; Fréard et al., 2007; Ba and Zhang, 2011; Paubel, 2011).

**Table 1:** Advantages of workload questionnaires.

Advantages	SWAT	WP	NASA-TLX	RSME	DALI
- Several dimensions leading to complementary information about workload	✓	✓	✓		✓
- Multidimensional workload: task demand, effort and performance			✓		✓
- Assesses the level of workload in a multimodal system (visual, auditory, etc.)		✓			✓
- Sensitive to the task difficulty		✓		✓	
- Assesses the subjective cost to perform a task			✓	✓	
- Compares the level of workload for several tasks with a different difficulty		✓			
- Predicts the task performance			✓		
- Analyzes the cognitive demands for a task	✓	✓			
- Used in real complex tasks	✓	✓	✓		
- Sensitivity	✓	✓	✓		
- Diagnosticity	✓	✓	✓		
- Selectivity/validity	✓	✓	✓		
- Intrusiveness	✓	✓	✓		

Among them, the WP and the NASA-TLX present more advantages than the SWAT, RSME, and DALI. However, as certain dimensions of mental workload are privileged in the questionnaires, the experimenters should choose the most appropriate questionnaire for their study as a function of the specific dimensions that they want to focus on. To our knowing, no studies have demonstrated that they respect the reliability, the implementation requirements and the subject acceptability.

Therefore, the most adapted questionnaire should not only respect the criteria but it should also assess the dimensions of mental workload that fit with the study’s goal. These questionnaires are effective to analyze the influence of situation complexity on the subjective level of mental workload and on driving performance.

### EFFECT OF SITUATION COMPLEXITY

Several studies have been carried out with the aim of showing the variations of subjective mental workload and of driving performance depending on situation complexity (i.e., De Waard, 1991; Cnossen et al., 2000; Steyvers and De Waard, 2000). To do so, the experimenters have generally tested different levels of complexity as a function of the road design, the road layout and the traffic flow (taxonomy of situations complexity, see Fastenmeier, 1995; Fastenmeier and Gstalter, 2007), or as a function of a single task (low level of complexity) vs. a dual-task (high level of complexity). As expected, two studies revealed that the increase of situation complexity led to a subjective workload enhancement and to performance impairments. Indeed, from a single task of driving to a dual-task (driving and answering the phone), the Standard Deviations of the Lateral Position (SDLP) and the Standard Deviations of Steering Wheel (SDSTW) increased (De Waard et al., 2001). Similarly, respectively, driving on the three sections “straight road,” “oncoming traffic,” and “city” increased the SDLP (Baldauf et al., 2009).

Contrary to the expectations, two studies have shown that the increase of situation complexity provoked a decrease of subjective workload and a driving performance improvement. More precisely, compared to low complex situations without any lane marking or with a limited visibility of the lane markings, complex situations comprising more information to process, with lane markings (Steyvers and De Waard, 2000) or with a high visibility of the lane markings (Horberry et al., 2006) improved the performance, with a decrease of SDLP and SDSTW (Steyvers and De Waard, 2000), and with a decrease of SDLP and centerlines crossings (Horberry et al., 2006). Therefore, the lane markings constituting additional information were a clue to guide the driver. The supplementary elements supposed to increase the situations complexity can thus sometimes facilitate the information processing instead of adding a supplementary load.

Several studies did not reveal any effect of situation complexity on subjective workload, probably because the situations were not sufficiently complex for the participants who were all experienced drivers. Although the increase of situations complexity, taking the SDLP into account, the performance did not vary between a highway without any entrance or exit and a highway with entrances and exits (De Waard, 1991), or even improved from the ordinary road preceding or following the experimental road to the experimental road, i.e., road leading through an open moorland (Jessurun et al., 1993) or road leading through a forest (De Waard et al., 1995). In these moderately complex situations, an elasticity of the resources availability probably allowed increasing their mobilization without feeling any cost, which allowed correctly performing the task (Kahneman, 1973; de Waard, 1996). However, a study revealed performance impairments with more SDLP on a complex road near a noise barrier than on a low complex road without any noise barrier (Jessurun et al., 1990). That could be due to a high mobilization of resources which was not perceived but which did not allow a good performance.

Moreover, when the situation complexity increases, compensatory mechanisms can be implemented in order to lower the level of mental workload and to maintain a good performance. For instance, drivers can reduce their speed to have time to process the whole information. Indeed, four studies revealed that despite the increase of subjective workload, drivers managed to maintain their performance with few SDLP in single task and in dual-task (Cnossen et al., 2000; Di Stasi et al., 2010), and even improved their performance with a decrease of SDLP from a single task to a dual-task (Brookhuis et al., 1991).

The diversity of results shows that the situation complexity does not always lead to an increase of subjective workload with performance impairments although it is generally the case for experienced drivers in very complex situations, probably reflecting an overload. In the following paragraph we are interested in this effect of driving experience on subjective mental workload and performance.

### EFFECT OF EXPERIENCE

A study revealed that faced with unexpected pedestrian crossings, whatever the situation complexity (straight road vs. winding road vs. very winding road with oncoming traffic), early-trained drivers had a higher subjective workload than more experienced drivers. Nevertheless, for all the drivers, an increase of subjective workload provoked performance impairments with an increase in the number of collisions with the pedestrians who suddenly crossed the road (Paxion et al., 2013). Therefore, the driving automation acquired with practice does not always allow improving driving performance. Experienced drivers have to be flexible by quickly switching from their automatic driving to a controlled driving when it is needed. Another study showed that as expected, faced to critical situations of accidents, all the drivers had performance impairments with an increase of the number of collisions, of the SDLP and of the time to brake, with all the same a better performance for experienced drivers than for the novice ones. Experienced drivers anticipated more and earlier than novices with a more efficient compensatory strategy (a speed reduction; Damm et al., 2011). Moreover, always comparing novice and experienced drivers, only the latter ones estimated that they would adopt the speed reduction strategy with the situation complexity enhancement implemented into pictures by an “extra” element (De Craen et al., 2008). These results show that novice drivers probably undervalue the situation complexity.

Contrary to our hypothesis, another study showed that the subjective level of workload was neither influenced by the situation complexity (single task vs. dual-task), nor by the driving experience (Patten et al., 2006).

Generally, the situation complexity increased the subjective mental workload which decreased with driving experience and led to performance impairments sometimes less observed for experienced drivers than for the novice ones. However, only few studies have tested the effect of situation complexity and experience on subjective mental workload and driving performance. The different physiological measures correlated to mental workload will now be described.

## PHYSIOLOGICAL MEASURES CORRELATED TO MENTAL WORKLOAD

### PHYSIOLOGICAL INDICATORS

The objective assessment of mental workload needs to take some of the subjective mental workload criteria into account (sensitivity, diagnosticity, intrusiveness, and reliability), as well as the generality of application, i.e., in laboratory and in operational environment (Kramer, 1991). Physiological indicators can advantageously complete subjective data. They allow a continuous on-line assessment that relatively quickly responds to phasic shifts in mental workload, even if the reaction latency depends on the measures. They are thus an indirect measure, correlated to mental workload. Moreover, they are non-intrusive, generally applicable in operational environments with a control of other factors that could influence the signal, such as the temperature, the light, etc. They can also be recorded in the absence of behavior as a baseline, and they provide a fine-grained analysis with a specific sensitivity to different mental workload dimensions (Kramer, 1991).

However, physiological indicators also present some limits. They are not entirely reliable for several reasons, i.e., different results are found depending on the studies, their interpretation requires a technical expertise (Kramer, 1985; Kramer, 1991), the discrimination between signal and noise is difficult when they both occur in the same frequency and time, and other factors than mental workload can influence the signal (e.g., physical exertion, emotional state, and ambient lighting; Kramer, 1991).

The following physiological measures are the most used to objectively assess the mental workload.

The electrocardiogram (ECG; the most common used) records the heart’s electrical activity which is necessary for the cardiac muscle’s contractions. Two main indicators are identified as being sensitive to mental workload (Mulder, 1986, 1988, 1992; de Waard and Brookhuis, 1991; Wilson and Eggemeier, 1991; Jorna, 1992; Boutcher and Boutcher, 2006; Brookhuis and De Waard, 2010; Causse et al., 2011; Gabaude et al., 2012). First, the mean Heart Rate (HR) refers to the number of beats per minute and a Differential or incremental HR (DHR) is also taken into account to precise the difference between two times, generally a rest period and an activity period. Second, the HR Variability (HRV) is defined by the variability of Inter-Beat Interval (IBI), i.e., the time duration (ms) between two consecutive peaks characterizing heartbeats. The HRV can be divided into several frequencies and the center of the mid frequency band (0.10 Hz component of HRV) is specifically used to identify the level of mental effort (de Waard, 1996).

The mental workload enhancement increases HR and DHR whereas it decreases HRV, especially in the 0.10 Hz band (Mulder et al., 2004; Brookhuis and De Waard, 2010). However, these indicators present some limits. The HR is not exclusively sensitive to changes in mental workload. It also reflects energetic, thermoregulatory, respiratory, emotional processes (Nickel and Nachreiner, 2003) as emotional strain, and physical activity (Jahn et al., 2005). The HRV does not always discriminate the level of difficulty, as in the study of Gabaude et al. (2012) that reveals no difference between a single task condition and a double task condition. The 0.10 Hz component of HRV also reveals changes in emotional strain and arousal and seems to be insufficient to assess the mental workload sensitivity (different levels of task difficulty) and diagnosticity (different types of tasks; Nickel and Nachreiner, 2003).

The electroencephalogram (EEG) records two types of indicators: bands of frequencies and event-related potentials (ERPs). Concerning the bands, the decrease in alpha band (8 to 13 Hz) and the increase in theta band (4 to 8 Hz) indicate an increase of mental workload (Kramer, 1991; Borghini et al., 2014) although more research is needed to precise this link (de Waard, 1996). Concerning the ERPs, they provide a picture of mental chronometry, especially distinguishing perceptual, cognitive and motor processes implicated in complex situations. Several long-latency ERP components (positive or negative potentials occurring 100, 200, or 300 ms after the stimulus presentation) are taken into consideration. The increase of onset latencies of some components (Ying et al., 2011), and the decrease of others (Miller et al., 2011) reveal a variation of mental workload. Generally, the amplitude of the P300 (or P3) component is the most often used (Brookhuis and De Waard, 2010). ERPs have a high diagnosticity to perceptual and cognitive processing, but they are insensitive to response factors, and they have a poor signal-to-noise ratio as they are influenced by other electrical signals (e.g., heart, eyes, muscles, and external sources; de Waard, 1996).

The electrodermal activity (EDA) records the autonomic changes in the electrical properties of the skin. Its sensitivity to mental workload variations is manifested with a positive correlation (Wilson, 2001; Chapon and Gabaude, 2009). It is often used as an indirect indicator of cognitive effort (Critchley et al., 2000) as it is not very selective and sensitive to various factors such as the respiration, temperature, humidity, arousal, and emotions (de Waard, 1996).

The ElectroOculoGram (EOG) records the eye activity. The increase of saccadic responses and peaks of saccadic velocity is interpreted as revealing a high level of mental workload in complex situations (Di Stasi et al., 2009). However, eye activity is probably more dependent on visual demands than on cognitive demands (de Waard, 1996).

Finally, the salivary sample of cortisol hormone can also be used. The increase of cortisol awakening response activates the hypothalamic-pituitary-adrenocortical (HPA) axis and reflects an increase of mental workload (Chida and Steptoe, 2009). Indeed, the cortisol rate indicates the level of stress and indirectly the effort provided to cope with it.

Therefore, the most sensitive measure to mental workload seems to be the ECG, although the choice should depend on the level of analysis, i.e., the fine-grained required for the mental workload assessment.

### EFFECT OF SITUATION COMPLEXITY

The following studies have analyzed the effects of situation complexity on objective mental workload and performance. As the ECG measure is the most commonly used, we only present studies using this technique which is highly correlated to mental workload. Different pattern of results have been found.

The situation complexity had an effect on objective mental workload with an increase of both mean HR (Liu and Lee, 2006; Collet et al., 2009; Mehler et al., 2011; Reimer et al., 2011) and incremental HR (Liu and Lee, 2006) when the complexity increased from a single task to a dual-task. Most of these complex situations studied above impaired the driving performance with an increase of SDLP (Reimer et al., 2011) and an increase of SD of Steering-Wheel angle (SDSTW; Liu and Lee, 2006), even when a compensatory strategy (speed reduction) was adopted (Liu and Lee, 2006). In the complex situations, the long and serial processing of the information probably impaired the driving activity with a lack of vehicle control (high SDLP and SDSTW). Concurrently to the increase of mean HR, performance impairments to the dual-task were observed with a decrease of the correct answer rates to the secondary task (Mehler et al., 2011) and longer reaction times to the dual-task than to the single one (Collet et al., 2009). The long reaction times could attest that complex situations require a controlled processing of the information, necessary for the decision-making and the maneuvers (tasks of the first and second level, see Michon, 1985). However, we need to nuance this interpretation, as Watson and Strayer (2010) have identified “supertaskers” who can manage to simultaneously perform several tasks without any performance decrement. Therefore, it is possible that some drivers correctly perform multitasks in very complex situations.

Contrary to expectations, HRV and its 0.10 Hz component were not impacted in the same way. Indeed, the situation complexity did not have any effect on HRV (Mehler et al., 2011) or only increased it (inverted value for compatibility reasons) in work periods compared to rest periods (Nickel and Nachreiner, 2000), revealing an increase of objective mental workload only between large levels of complexity. It’s probably because HRV is not sufficiently sensitive to low differences of task difficulty’s levels. Moreover, the 0.10 Hz component of HRV did not always vary between the different types of tasks (Nickel and Nachreiner, 2000), and even sometimes revealed a decrease of mental effort from a single task to a dual-task (Nickel and Nachreiner, 2003). However, the results showed better performance to easy tasks than to the difficult ones with shorter reaction times and less errors (Nickel and Nachreiner, 2000, 2003). These results confirm that the 0.10 Hz component of HRV does not assess the diagnosticity of mental workload in these studies, and probably does not exclusively assess the level of mental workload. The levels of emotional strain and arousal have also probably influenced this component. Moreover, short reaction times to easy tasks certify that they are rapidly performed, probably due to an automatic processing of the information.

These studies confirm the positive relation between the situation complexity and the physiological measures correlated to mental workload. However, this relation was only observed with the mean HR and the incremental HR. The situation complexity also led to performance degradations. As far as we know, no study has considered the effect of experience on physiological measures of mental workload without considering the subjective level of workload. In the following paragraph, we thus present studies comparing the effect of situation complexity and experience on subjective and objective measures of mental workload.

## COMPARISONS BETWEEN SUBJECTIVE AND PHYSIOLOGICAL MEASURES

### EFFECT OF SITUATION COMPLEXITY

In the study of Dijksterhuis et al. (2011), the increase of situation complexity (decreasing lane widths) only increased the subjective effort but neither the mean HR nor the HRV. Contrary to expectations, the complexity enhancement improved the performance with a better control of the lateral position of the vehicle. Therefore, the complex situation helped the drivers to control their trajectory although they probably overvalued their internal state. In the same study, with another type of situation complexity (from low to high oncoming traffic density), an increase of subjective effort, a decrease of HR and an increase of HRV were observed. The performance was also improved with a decrease of the SDLP. Therefore, the drivers might have overvalued their effort, probably because they had to process more information with a high density of oncoming traffic, but they did not need to make a physiological effort to control their trajectory. Other studies have also shown that the increase of situation complexity increased the subjective mental workload but decreased the objective mental workload with an increase of the IBI and the 0.10 Hz component of HRV (Brookhuis et al., 2008), and an increase of HRV without any effect on HR (Gabaude et al., 2012). These results reveal that drivers generally overvalued their mental workload. They probably felt a cost to realize the task although their internal state was not modified.

In another study (De Waard et al., 2009), the increase of situation complexity (heavy goods vehicles enhancement) had a different impact on mental workload and performance depending on the driving section. In the acceleration lane, during the merging into traffic and when exiting traffic, the complexity enhancement increased the subjective mental effort but not the mean HR nor the 0.10 Hz component of HRV. Drivers systematically adopted the compensatory strategy of the average speed reduction. This strategy had different effects on performance as a function of the section. In the acceleration lane, that allowed them maintaining a good performance with a better control of the lateral position and the speed. When exiting traffic, drivers ensured a better control of speed and a non-reduction of safety margins. During the merging into traffic, performance degradations were observed with a poor control of the lateral position and a reduction of safety margins increasing the risk of accident. During the section of the lane change maneuver before exiting traffic, the complexity had no effect on the subjective effort but increased the mean HR and decreased the 0.10 Hz component of HRV. Therefore, the same complexity can require more or less mental workload which is felt differently as a function of the type of driving sections. Moreover, a high complexity introduced before merging into traffic did not increase the subjective or objective levels of mental workload while the merging, but drivers reduced the safety margins. Conversely, a low complexity reduced the subjective mental effort and unexpectedly, the compensatory strategy of reducing the speed did not improved driving performance with a poor control of the lateral position and a reduction of safety margins (De Waard et al., 2009). Therefore, the merging task should have been difficult whatever the previous situation.

Several studies showed that a high complexity of driving situations increased the subjective and objective levels of mental workload, indicating that drivers had a right assessment of their physiological state. However, most of the studies revealed that the increase of situation complexity only increased the subjective level of mental workload, showing that drivers overvalued their objective mental workload. Moreover, the performance was often improved, probably because the supplementary difficulty of the task resulted in a higher concentration without providing a lot of resources. These studies were carried out on experienced drivers but a difference with novice drivers should appear.

### EFFECT OF EXPERIENCE

de Waard et al. (2008) showed that for novice and experienced drivers, the increase of situation complexity for the task of merging into traffic increased the subjective mental effort but did not have any effect on the mean HR and even decreased the objective mental workload with an increase the 0.10 Hz component of HRV. This could reflect resilience to the situation assessed as too difficult to be able to compensate by making a physiological effort although an effort was felt, probably due to the situation complexity. Nevertheless, compared to before and after merging into traffic, the merging increased the objective mental workload with a mean HR enhancement and a decrease of the 0.10 Hz component of HRV. This could be explained by a large difference of difficulty with a high level during vs. a low level before and after the merging maneuver. Generally, drivers slowed down but their performance was impaired with an increase of speed variation and a decrease of safety margins. Experienced drivers had especially more variations in speed than inexperienced drivers. The absence of a high distinction between novice and experienced drivers is probably due to the fact that in the experiment, inexperienced drivers had already around 2 years of experience.

With a sample of novice drivers, the increase of situation complexity did not have any effect on subjective effort nor on HRV although it shortened the IBI. Novice drivers thus probably undervalued their mental workload, which led to performance impairments in the dual-task condition, i.e., in the complex situation (Veltman and Gaillard, 1996).

In summary, the increase of situation complexity always impaired novice drivers’ performance, even if they overvalued or undervalued their mental workload. Nevertheless, few studies comparing novice drivers to more experienced ones have been carried out. It is thus important to qualify these results.

## DISCUSSION

First, this paper aimed to indicate how to choose the most adapted subjective measure of mental workload and to verify the main hypothesis which is that the increase of situation complexity and the lack of experience increase the subjective level of mental workload and lead to driving performance impairments. Among the studies indexed in this article, the questionnaires assessing the subjective mental workload which are the most used are the RSME (46%), the NASA-TLX (23%), the SWAT (4%) and the DALI (4%), with 23% of less known questionnaires. Although the NASA-TLX and the WP are consistent with the majority of the required criteria, the RSME is the most used in the studies presented in this paper. The choice of questionnaire thus also depends on the context of the study and on the mental workload dimensions that the experimenter wants to focus on.

Concerning the link between situation complexity and subjective mental workload, we need to be cautious about the definition of a complex situation. Indeed, the literature shows that the increase of information to process produces a supplementary difficulty but it can also help the drivers by serving as a clue (e.g., presence of lane markings). Contrary to what we could imagine, the quantity of information to process thus does not define the situation complexity. It is thus rather characterized by different elements (see the taxonomy, Fastenmeier, 1995; Fastenmeier and Gstalter, 2007). The studies carried out on experienced drivers have shown different pattern of results. Generally, only very complex driving situations increased the subjective workload and impaired the performance, confirming our hypothesis. Drivers were thus probably overwhelmed by the very complex situation, indicating an overload. In the situations probably assessed as moderately complex by the experienced drivers, they often adopted the compensatory strategy of reducing their speed, which probably helped them to correctly perform the driving task. Among the few studies that have compared novices to experienced drivers, the increase of situation complexity increased the subjective mental workload for all the drivers but with a lower level for the experienced ones. As expected, the increase of situation complexity also led to performance impairments sometimes lower for the experienced drivers than for the novice ones. It is probably because experienced drivers have more automatized the driving tasks, even those requiring a high-level processing, i.e., tasks of the second level (tactical) of the tasks hierarchy’s model (Michon, 1985; de Waard, 1996). However, studies only observing the physiological measures of mental workload without the subjective level of workload are needed to verify our hypothesis.

Second, the aim of this paper was to identify the most representative physiological measures correlated to mental workload, and to review the empirical studies showing the effect of situation complexity and driving experience on these physiological measures and on driving performance. Among the physiological indicators, the mean HR and the incremental HR seem to have the most advantages to be correlated to mental workload. It is confirmed by the studies that have mainly found significant results with these two indicators, contrary to the HRV and the 0.10 Hz component of HRV that not seem to be sensitive enough to different levels of complexity. Indeed, only considering the mean HR and the incremental HR, the reviewed studies globally confirmed that very complex situations increased physiological measures correlated to mental workload and impaired driving performance. The physiological interpretation thus needs to be made with caution as these measures can reflect different factors. The question is thus not only to choose the adequate measure but it is also to choose the best subjective and objective complementary measures. Indeed, physiological measures respond to disadvantages of subjective measures (e.g., an off-line method, with recall and post-rationalization biases). Reciprocally, subjective measures respond to some disadvantages of physiological measures (e.g., a long and technical process of analysis, and a low selectivity with a high sensitivity to various factors).

Finally, the third aim of this paper was to verify whether the increase of situation complexity and the lack of experience increase the subjective and physiological levels of mental workload and lead to driving performance impairments. When observing the effects of both subjective and objective mental workload of experienced drivers, the situation complexity mainly increased the subjective level of mental workload but not the physiological state, and often improved the performance. Experienced drivers were not always aware of their internal state which was mostly overvalued. Moreover, they probably felt a high effort because they were concentrated on the task which was performed with success. Concerning the novice drivers, the increase of situation complexity only provoked an increase of the subjective level or of the physiological measures, with performance impairments. Novices thus either overvalued or undervalued their objective mental workload. When they undervalued their state, they probably did not adopt any compensatory strategy, which have made the task difficult to perform. When they overvalued their state, they probably also overvalued their abilities because of the lack of experience (optimism bias, see McKenna, 1993), which led them to not adopt compensatory strategies. Other studies analyzing both subjective and objective mental workload need to be carried out in order to precise the impacts on driving performance.

Nowadays, few studies have observed the combined effects of situation complexity and driving experience on mental workload and driving performance and these effects must be highlighted. Indeed, it would be useful to target the driving learning as a function of the learners’ assessment about the complexity of the situation and about their mental and behavioral abilities, knowing that their assessment can change with driving experience. As soon as they have their driving license, novices could adapt their driving depending on the situation complexity and on their state as they would know how to manage the task with an awareness of many parameters. Thus, an experimental study testing the effect of situation complexity and driving experience on the subjective and physiological levels of mental workload and on performance could identify all the relationships between these factors of accidents among the young drivers.

## Conflict of Interest Statement

The authors declare that the research was conducted in the absence of any commercial or financial relationships that could be construed as a potential conflict of interest.
